# First description of the male of *Meotipa vittiforma* (Araneae, Theridiidae), with a discussion of its palpal morphology

**DOI:** 10.3897/BDJ.14.e191299

**Published:** 2026-04-30

**Authors:** Haosiyi Zhu, Guolong Huang, Jie Liu, Changhao Hu

**Affiliations:** 1 Arachnid Resource Centre of Hubei Province & Hubei Key Laboratory of Regional Development and Environmental Response, Faculty of Resources and Environmental Science, Hubei University, Wuhan, China Arachnid Resource Centre of Hubei Province & Hubei Key Laboratory of Regional Development and Environmental Response, Faculty of Resources and Environmental Science, Hubei University Wuhan China; 2 Centre for Behavioral Ecology and Evolution, School of Life Sciences, Hubei University, Wuhan, China Centre for Behavioral Ecology and Evolution, School of Life Sciences, Hubei University Wuhan China; 3 College of Life Sciences, Hunan Normal University, Changsha, China College of Life Sciences, Hunan Normal University Changsha China

**Keywords:** biodiversity, canopy, cobweb spiders, male palp, taxonomy

## Abstract

**Background:**

The genus *Meotipa* Simon, 1895 contains 33 extant species, 18 of which are known from China. However, no detalied morphological study of the expanded male palp in this genus has been conducted. *Meotipa vittiforma* Hu, Zhong, Liu & Li, 2026 was originally described, based on a female specimen collected from Hainan Province, China and its male remains unknown.

**New information:**

The male of *Meotipa vittiforma* Hu, Zhong, Liu & Li, 2026 was identified and described for the first time herein based on the specimens collected from the forest canopies of Hubei Province, China. In addition, we discuss several unique characters of its male palp that have not been previously recorded in the "lost colulus clade".

## Introduction

*Meotipa* Simon, 1895 was first mentioned by Simon (1894) based on two species from India and the Philippines. However, because the genus was introduced without a description or definition, the name initially constituted a nomen nudum (ICZN 1999: Article 12). Simon (1895) subsequently provided a description, thereby validating the generic name. Levi and Levi (1962) treated *Meotipa* as a junior synonym of *Chrysso* O. Pickard-Cambridge, 1882, but the genus was re-validated by Deeleman-Reinhold (2009). Deng et al. (2022) reviewed the species of *Meotipa* from China and described two new species from Yunnan Province. Hu et al. (2026b) revised the *Chrysso* sensu lato, describing two new species and transferring four species from *Chrysso* to *Meotipa*. At present, 33 species of *Meotipa* have been reported from eastern, southern and south-eastern Asia, with only one species known from Pacific Islands and introduced to Africa and Americas (World Spider Catalog 2026). A distinctive morphological feature of *Meotipa* spiders is the presence of flattened black spines on the opisthosoma and legs. They usually inhabit the underside of leaves and exhibit egg sac and larva guarding behaviour (Deeleman-Reinhold 2009; personally observation).

Forests canopies represent the aggregate of all crowns in a forest stand and support about 40% of extant species (Ozanne et al. 2003). A survey of Arthropoda Gravenhorst, 1843 in tropical to subtropical forests of China conducted by colleagues from the Chinese Academy of Sciences, Hubei University and Shenyang Normal University has resulted in the discovery of several new taxa (e.g. Hu and Liu (2025)).

*Meotipa vittiforma* Hu, Zhong, Liu & Li, 2026 was originally described by Hu et al. (2026b), based on a female specimen collected from Hainan Province, China. During the examination of theridiid spiders collected from Hubei Province, China, *M. vittiforma* was identified together with its previously unknown male. Through the expansion and detailed observation of the male palp, several unusual characters were observed, including the tibial rim and the tegular apophysis (Hu et al. 2026a). The aims of the current paper are to describe the male of *M. vittiforma* for the first time, to provide a new provincal record of this species and to discuss the morphology of its male palp.

## Materials and methods

The specimens examined in this study are deposited in the Centre for Behavioral Ecology and Evolution (CBEE), School of Life Sciences, Hubei University in Wuhan, China (curator: Jie Liu). Specimens were examined using a LEICA M205 C stereomicroscope. The male palp was examined and photographed after dissection. For expansion, the male palp was immersed in lactic acid and heated in a water bath at 56℃. The epigyne was dissected from the body, immersed in 0.1mg/ml Protease K solution and heated in a water bath at 56℃. All measurements were taken using a Leica M205 C stereomicroscope. Eye diameters were taken at the widest point. Leg measurements are given as the total length of leg (femur, patella, tibia, metatarsus, tarsus). All measurements are in millimetres (mm). Photographs were taken using an OLYMPUS SXZ16 microscope and multifocal images were produced using Helicon Focus (Version 7.7.0). The distribution map was generated using ArcGis (Version 10.8.1) (ESRI 2011). Terminology used in the text and figures follows Agnarsson et al. (2007).

Abbreviations: A–atrium; ALE–anterior lateral eye; AME–anterior median eye; C–conductor; CD–copulatory duct; CH–cymbial hood; CO–copulatory opening; CP–conductor distal projection; E–embolus; FD–fertilisation duct; MA–median apophysis; P–pocket; PLE–posterior lateral eye; PME–posterior median eye; S–spermatheca; SD–sperm duct; ST–subtegulum; T–tegulum; TA–tegular apophysis; Tb–trichobothrium; I, II, III, IV–legs I to IV.

## Taxon treatments

### Meotipa vittiforma

Hu, Zhong, Liu & Li, 2026

54371168-875A-56CE-8D58-2AD485783924

*Meotipa vittiforma Hu et al. 2026b*: 204, figs. 5C, D, 7D, H, L (description of female).

#### Materials

- **Type status:** Holotype. **Occurrence:** recordedBy: Fengxiang Liu and Zichang Li; individualCount: 1; sex: female; lifeStage: adult; **Taxon:** scientificName: *Meotipa vittiforma* Hu, Zhong, Liu & Li, 2026; namePublishedIn: Hu C. H., Zhong R., Liu J., and Li Z. C. 2026. ZooKeys, 1266:204; kingdom: Animalia; phylum: Arthropoda; class: Arachnida; order: Araneae; family: Theridiidae; genus: *Meotipa*; taxonRank: species; **Location:** country: China; countryCode: CN; stateProvince: Hainan Province; locality: Diaoluoshan; verbatimElevation: 136 m; verbatimLatitude: 18.7814° N; verbatimLongitude: 109.5161° E; **Event:** year: 2018; month: 3; **Record Level:** institutionID: CBEE; datasetID: LJ201800979
- **Type status:** Other material. **Occurrence:** recordedBy: Hailun Chen, Guolong Huang and Yunhe Wang; individualCount: 2; sex: 1 male, 1 female; lifeStage: adult; **Taxon:** scientificName: *Meotipa vittiforma* Hu, Zhong, Liu & Li, 2026; namePublishedIn: Hu C. H., Zhong R., Liu J., and Li Z. C. 2026. ZooKeys, 1266:204; kingdom: Animalia; phylum: Arthropoda; class: Arachnida; order: Araneae; family: Theridiidae; genus: *Meotipa*; taxonRank: species; **Location:** country: China; countryCode: CN; stateProvince: Hubei Province; county: Enshi Tujia and Miao Autonomous Prefecture, Hefeng County; locality: Pingshan Canyon; verbatimElevation: 650 m; verbatimLatitude: 29.9285° N; verbatimLongitude: 110.0681° E; **Identification:** identifiedBy: Changhao Hu; identificationReferences: Hu et al. 2026; **Event:** year: 2024; month: 6; day: 18; **Record Level:** institutionID: CBEE; datasetID: PWHB2024020, 2024021

#### Description

**Male** (first description, PWHB2024020): Total length 2.36; carapace length 1.11, width 0.91; opisthosoma length 1.25, width 0.79; sternum length 0.63, width 0.51. Eyes: AME 0.35, ALE 0.31, PME 0.36, PLE 0.35, AME–AME 0.32, AME–ALE 0.13, PME–PME 0.22, PME–PLE 0.21 AME–PME 0.27, ALE–PLE 0.04. Measurements of leg [legs I and IV missing]: II 8.63 (2.58, 0.57, 1.90, 2.50, 1.08), III 7.12 (2.34, 0.30, 1.64, 1.86, 0.98).

**Figure 1.**
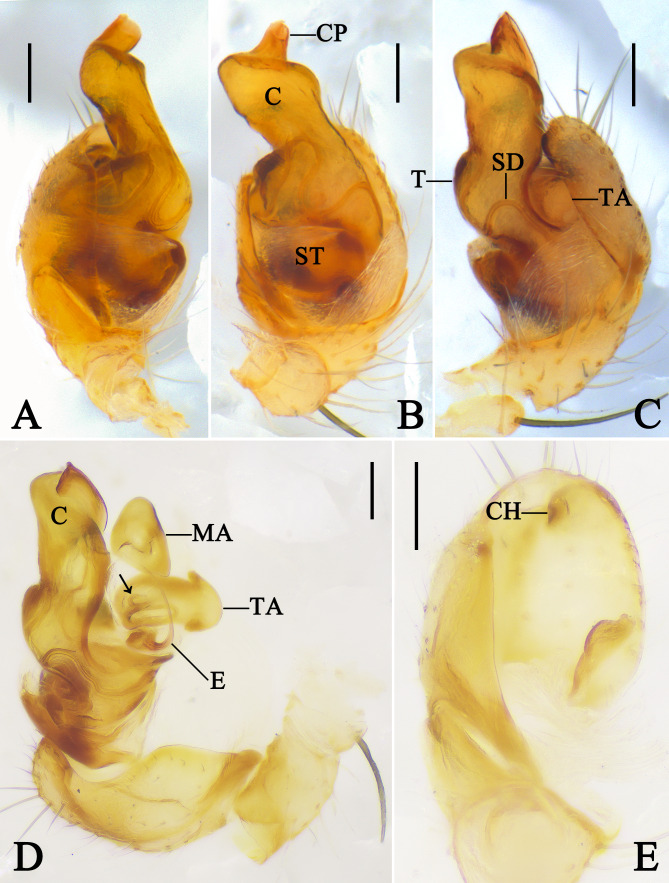
Left male palp of *Meotipa vittiforma* Hu, Zhong, Liu & Li, 2026 (PWHB2024020). **A** Prolateral view; **B** Ventral view; **C** Retrolateral view; **D** Expanded, retrolateral view; arrow points to the sperm duct inside the tegular apophysis; **E** Cymbium, prolateral view. Abbreviations: C–conductor; CH–cymbial hood; CP–conductor distal projection; E–embolus; MA–median apophysis; SD–sperm duct; ST–subtegulum; T–tegulum; TA–tegular apophysis. Scale bars = 0.1 mm.

**Figure 2.**
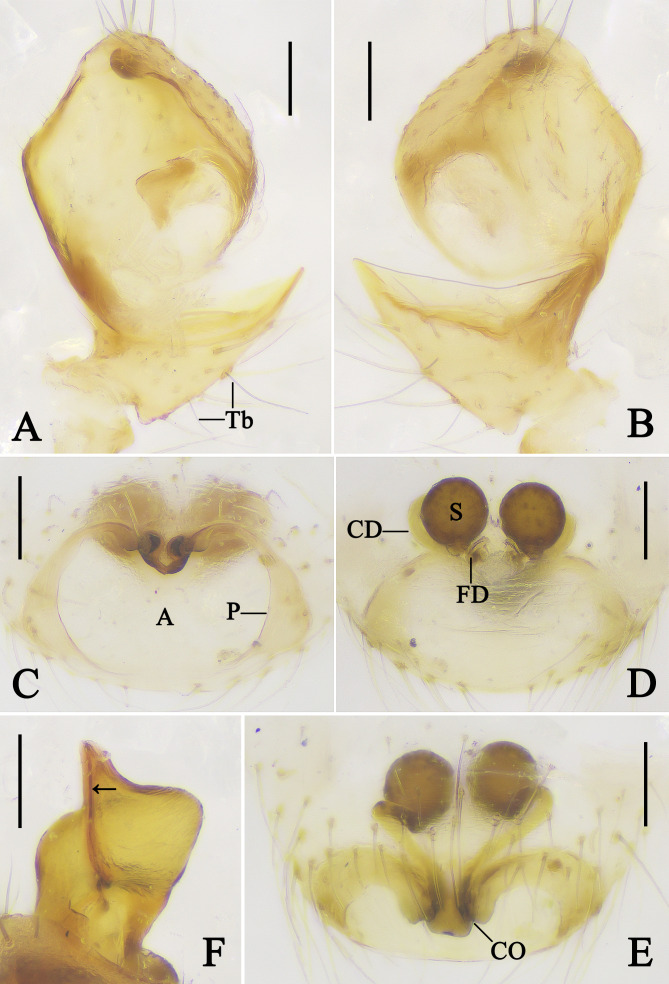
Copulatory organs of *Meotipa vittiforma* Hu, Zhong, Liu & Li, 2026 (A, B, F. PWHB2024020; C–E. PWHB2024021). **A** Tibia and cymbium of left male palp, ventral view; **B** Tibia and cymbium of left male palp, dorsal view; **C** Epigyne, ventral view; **D** Vulva, dorsal view; **E** Epigyne, antero-ventral view; **F** Conductor, dorsal view; arrow points to the ridge. Abbreviations: A–atrium; CD–copulatory duct; CO–copulatory opening; FD–fertilisation duct; P–pocket; S–spermatheca; Tb–trichobothrium. Scale bars = 0.1 mm.

Palp (Figs 1, 2A, B and F): Patella retrolaterally with a spine. Tibia almost triangular, retrolaterally with two trichobothria; tibial rim facing the dorsum of cymbium. Cymbium almost quadrangular in ventral view. Cymbial hood tiny, situated anterior part of cymbium. Subtegulum bowl-shaped. Tegulum as wide as bulb, with sperm duct M-shaped in ventral view. Tegular pit absent. Median apophysis almost triangular. Tegular apophysis almost as large as median apophysis and arrow-shaped, inside with sperm duct (arrow in Fig. 1D). Conductor sclerotised, almost 2/3 the width of the tegulum, distal part with a projection and dorsally with a ridge (arrow in Fig. 2F). Embolus needle-like and curved.

**Figure 3.**
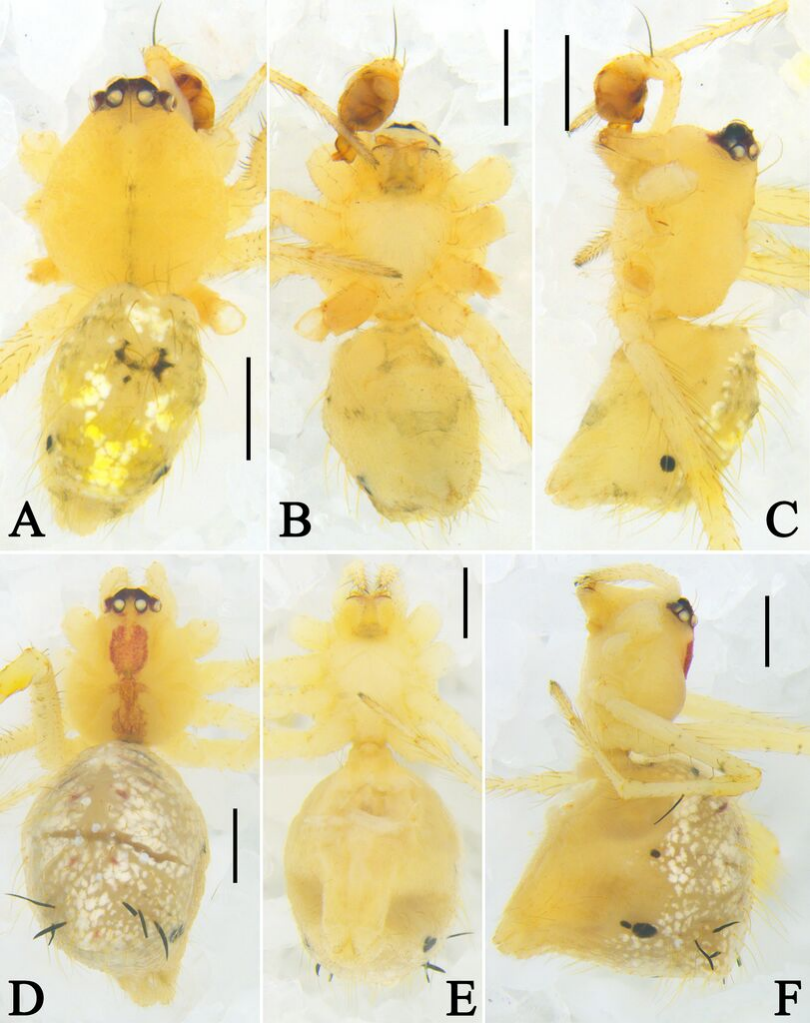
Habitus of *Meotipa vittiforma* Hu, Zhong, Liu & Li, 2026. **A–C** Male (PWHB2024020); **D–F** Female (PWHB2024021). **A, D** Dorsal view; **B, E** Ventral view; **C, F** Lateral view. Scale bars = 0.5 mm.

Habitus (Fig. 3A–C): Carapace pear-shaped, yellow, medially with a slightly black longitudinal line, eye region slightly red. Sternum, chelicerae, labium and spinnerets yellow. Endites yellow with black distal margins. Palps and legs yellow and haired. Opisthosoma pale yellow; dorsum haired, anteriorly with white and black markings, medially with a V-shaped pattern consisting of white and yellow patches; posterior extension laterally with a large black spot.

**Female** (redescription, PWHB2024021): Total length 2.77; carapace length 1.06, width 0.91; opisthosoma length 1.71, width 1.27; sternum length 0.71, width 0.57. Eyes: AME 0.46, ALE 0.40, PME 0.46, PLE 0.41, AME–AME 0.52, AME–ALE 0.08, PME–PME 0.34, PME–PLE 0.27 AME–PME 0.38, ALE–PLE 0.05. Measurements of leg [legs I, II missing]: III 6.26 (2.01, 0.33, 1.32, 1.76, 0.84), IV 10.62 (3.55, 0.65, 2.39, 3.05, 0.98).

Epigyne (Fig. 2C–E): Epigynal field wider than long, with an atrium almost 1.5 times wider than long, laterally with triangular pockets, anterior margins of atrium lingulate, with two notches, copulatory openings situated in the notches. Copulatory ducts V-shaped in anterior view. Spermathecae spherical. Fertilisation ducts arising from posterior position of spermathecae.

Habitus (Fig. 3D–F): Generally, as in male. Carapace medially with a red longitudinal band. Legs with flattened black spines. Dorsal part of opisthosoma full of white patches, anteriorly with black and red transverse lines; laterally with three black spots, one large and two small; posterior extension with flattened black spines.

#### Diagnosis

The male of *M. vittiforma* is similar to that of *M. lingchuanensis* (Zhu & Zhang, 1992) in having a wide conductor with distal projection (cf. Fig. 1A–C and fig. 6 in Hu et al. (2026b)), but can be recognised by: 1) sperm duct inside retrolateral part of tegulum curved (straight in *M. lingchuanensis*); 2) the extended beyond cymbium part of conductor almost half the length of cymbium (almost 1/4 in *M. lingchuanensis*); and 3) distal projection of conductor digitiform (hook-like in *M. lingchuanensis*). For female, see Hu et al. (2026b).

#### Distribution

**Figure 4.**
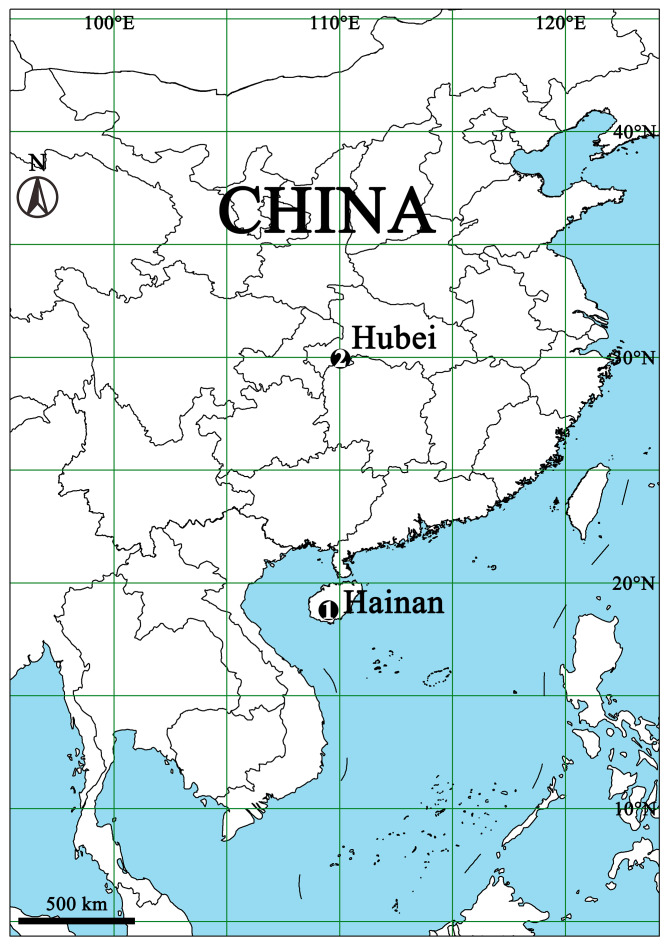
Distribution map of *Meotipa vittiforma* Hu, Zhong, Liu & Li, 2026. **1** Type locality; **2** The location in the current paper.

China (Hainan, type locality; Hubei, new provincal record) (Fig. 4).

## Discussion

Agnarsson et al. (2007) discussed the morphological landmarks and terminology of the theridiid male palp. During our examination of the specimen in the current study, several unusual characters were observed:

**1) Tibial rim facing the dorsal cymbium**.

Agnarsson et al. (2007) noted that the tibial rim always faces the palpal bulb within the cymbium. In the character matrix for character 17 in Agnarsson (2004), most theridiids exhibit a tibial rim facing the bulb, except for the genus *Phoroncidia* Westwood, 1835 and the subfamily Hadrotarsinae (*Euryopis* Menge, 1868, *Dipoena* Thorell, 1869, *Emertonella* Bryant, 1945). In *M. vittiforma*, the tibial rim faces the dorsal cymbium (Fig. 2B), representing a rare state within the “lost colulus clade” to which the genus *Meotipa* belongs.

**2) Median apophysis lacking sperm duct, whereas the tegular apophysis has sperm duct**.

Agnarsson et al. (2007) defined the median apophysis as a structure forming part of the theridiid bulb-cymbium locking mechanism and noted that it may contain a loop of sperm duct. Based on its mechanical relationship with the cymbial hood, we recognise the triangular sclerite as the median apophysis. The absence of sperm duct inside the median apophysis is common within the “lost colulus clade”.

The tegular apophysis is typically located close to the embolus in the topological structure of the palp. The positional relationship observed here, together with the exclusion of the above-mentioned median apophysis, suggests that the arrow-shaped sclerite is the tegular apophysis (Agnarsson et al. 2007). However, we observed that the tegular apophysis has a sperm duct (Fig. 1D), which is unusual in theridiids. Coddington (1990) applied the term “TTA (theridiid tegular apophysis)” to the sclerite bearing sperm duct, but this viewpoint was rejected by Agnarsson et al. (2007): “He applies it to the MA whenever the MA had sperm ducts going through it, but to the ‘true’ TTA when the MA was without ducts”.

This study provides the first detailed description of the expanded male palp in the genus *Meotipa*. In the unexpanded palp, the wide tegulum and conductor cover most sclerites, making them difficult to observe. Murthappa et al. (2017) concluded that some *Meotipa* species lack apophysis in the palp. However, our observations show that all five major sclerites (subtegulum, tegulum, median apophysis, tegular apophysis, embolus) of the bulb are present in *M. vittiforma*. These distinctive characters may indicate the existence of an independent evolutionary lineage within Meotipa (Murthappa et al. 2017).

## Supplementary Material

XML Treatment for Meotipa vittiforma
